# Neonatal injury models: integral tools to decipher the molecular basis of cardiac regeneration

**DOI:** 10.1007/s00395-022-00931-w

**Published:** 2022-05-03

**Authors:** Alessia Costa, Sarah Cushman, Bernhard J. Haubner, Anselm A. Derda, Thomas Thum, Christian Bär

**Affiliations:** 1 Institute of Molecular and Translational Therapeutic Strategies (IMTTS), Hannover Medical School, Hannover, Germany; 2 REBIRTH-Centre for Translational Regenerative Medicine, Hannover Medical School, Hannover, Germany; 3 Department of Internal Medicine III (Cardiology and Angiology), Innsbruck Medical University, Innsbruck, Austria; 4 Department of Cardiology, University Heart Center, University Hospital Zurich, Zürich, Switzerland; 5 Department of Cardiology and Angiology, Hannover Medical School, Hannover, Germany; 6 Fraunhofer Institute for Toxicology and Experimental Medicine (ITEM), Hannover, Germany

**Keywords:** Cardiac regeneration, Cardiomyocyte proliferation, Myocardial infarction, microRNA, Neonatal heart injury, Regenerative medicine

## Abstract

Myocardial injury often leads to heart failure due to the loss and insufficient regeneration of resident cardiomyocytes. The low regenerative potential of the mammalian heart is one of the main drivers of heart failure progression, especially after myocardial infarction accompanied by large contractile muscle loss. Preclinical therapies for cardiac regeneration are promising, but clinically still missing. Mammalian models represent an excellent translational in vivo platform to test drugs and treatments for the promotion of cardiac regeneration. Particularly, short-lived mice offer the possibility to monitor the outcome of such treatments throughout the life span. Importantly, there is a short period of time in newborn mice in which the heart retains full regenerative capacity after cardiac injury, which potentially also holds true for the neonatal human heart. Thus, in vivo neonatal mouse models of cardiac injury are crucial to gain insights into the molecular mechanisms underlying the cardiac regenerative processes and to devise novel therapeutic strategies for the treatment of diseased adult hearts. Here, we provide an overview of the established injury models to study cardiac regeneration. We summarize pioneering studies that demonstrate the potential of using neonatal cardiac injury models to identify factors that may stimulate heart regeneration by inducing endogenous cardiomyocyte proliferation in the adult heart. To conclude, we briefly summarize studies in large animal models and the insights gained in humans, which may pave the way toward the development of novel approaches in regenerative medicine.

## Introduction

Cardiovascular diseases (CDVs) are a group of the most common, serious, chronic, and life-threatening disorders causing death, disabilities and huge socioeconomic costs globally. A common characteristic of most non-congenital heart diseases is the extensive loss of contractile cardiomyocytes. For example, severe MI can wipe out more than 25% of ventricular cardiomyocytes within a few hours [50]. Subsequently, inflammatory processes are induced, necrotic cardiomyocytes are phagocytized and replaced by myofibroblasts, which proliferate and synthesize collagen, thereby producing a fibrous scar [89]. From the traditional point of view, the heart is considered a post-mitotic organ, i.e., a terminally differentiated organ unable to renew dead cardiomyocytes. Textbooks usually state that “the heart survives and exerts its function until the death of the organism with the same or lesser number of cells that present at birth” [6]. In recent years, this theory has been refuted to some extent and it is now widely accepted that there is cardiomyocyte turnover in the adult heart. Although this turnover is insufficient to replace the cardiomyocytes lost following an ischemic event, it has triggered research aiming to understand and harness the underlying mechanisms of cardiac regeneration in mammals [11, 85]. In particular, the fact that cardiomyocytes are highly proliferative in neonatal hearts, but then withdraw from the cell cycle soon after birth raised several still unresolved questions: Can we induce proliferation to compensate for lost cardiomyocytes in the heart after an injury? Which cell populations are contributing to cardiomyocyte regeneration? When does cardiomyocyte proliferation stop and what are the underlying molecular cues?

Current strategies to replace lost cardiomyocytes rely on either the stimulation of endogenous regenerative processes, including the stimulation of cardiomyocyte proliferation and the conversion of other cardiac cell types, (e.g., transdifferentiation of fibroblasts into cardiomyocytes) [79], or the transplantation of stem/progenitor cells [40]. Some studies support the theory that the replacement of dead cells in the heart may rely on the ability of stem cells or progenitors to differentiate into cardiomyocytes [11]. Other studies instead indicate that the best potential for replacement of dead cardiomyocytes lies in pre-existing cardiomyocytes. The authors have previously demonstrated that cardiomyocyte turnover from pre-existing cardiomyocytes is approximately 0.76% per year in the young adult mouse under normal homeostatic conditions and this rate is seen to decreases with age [85]. Thus, the development of therapeutic approaches to stimulate endogenous regenerative processes and the potential to make these approaches translatable for the treatment of cardiac pathologies in human patients is of utmost importance.

## Cardiac regeneration potential: from invertebrates to mammals

The first evidence of complete regeneration after amputation is seen in lower clades of the animal kingdom. *Planarians, Annelids and Hydra* represent the group of invertebrates having a spectacular capacity to regenerate all tissues and organs by both tissue remodeling and through the proliferation of pre-existing and resident adult somatic stem cells. Additionally, some lower vertebrates such as newts, axolotls and teleost fish can regenerate fins, limbs and tails after traumatic damage [28, 71]. These discoveries provide crucial insights for the understanding of cellular and molecular mechanisms of organ restoration in vertebrates.

Recently, studies have demonstrated that amphibians and zebrafish also retain the ability to regenerate the heart even as adults. Notably, zebrafish (*Danio rerio*) can undergo complete cardiac regeneration without scar formation within 2 months after 20% apical resection of the ventricle through a robust proliferation of resident cardiomyocytes [42]. Similarly, zebrafish can also regenerate the heart also following cryoinjury, but the process is much slower comparably, taking around 180 days [35]. Interestingly, regeneration of a zebrafish heart appears fundamentally different from the processes described in other tissues. For example, in the fin of a zebrafish, a mass of pluripotent cells adjacent to the wound site (*blastema*) responds to the stimulus of an amputation. Differentiation of these cells results in new tissues functionally integrated with the pre-existing tissue [71]. In contrast, while epicardial progenitor cells may contribute to the generation of new cardiomyocytes, the main source for newly formed cardiomyocytes are pre-existing cardiomyocytes, which undergo partial de-differentiation and then re-enter the cell cycle [42].

The debate is still ongoing with regard to mammalian hearts, in which cardiomyocyte renewal is evident but certainly insufficient for the restoration of contractile function after cardiac damage. The differences in anatomy, biology and physiology between mammal and zebrafish hearts needs to be additionally considered when studying cardiac regenerative potential in higher vertebrates. First, zebrafish cardiomyocytes are small, mono-nucleated and able to proliferate [76]. Further, the number of heart chambers, four in mammals and two in zebrafish, and the kind of circulation, double circulation in mammals and single circulation in zebrafish with the mixing of arterial and venous blood, are other significant differences. More importantly, zebrafish reside in an aquatic environment that is hypoxic and may promote cardiomyocyte de-differentiation and proliferation [78]. Additionally, poikilothermic zebrafish have an anaerobic metabolism and lower oxygen consumption, attributed to a higher relative glycolysis capacity compared to other species. In contrast, mammals utilize mitochondrial respiration as a source of energy and require a high oxygen supply that is crucial for satisfying the metabolic demands for their vital processes [48]. Nevertheless, a sort of parallelism between the mammalian embryonic stage heart and the adult zebrafish heart exists. Anatomically, during cardio-genesis (before the septation phase), the embryonal mammalian heart is characterized by two primitive chambers which will develop into four chambers toward the end of the development of the heart [17]. Further similarities exist in terms of tissue, cellular composition and metabolic features.

Similar to zebrafish, embryonic and neonatal murine cardiomyocytes require lower oxygen for metabolic processes using glycolysis as a source of energy. Indeed, it is well documented that for a few days, the neonatal mouse heart retains full regenerative potential as demonstrated in the apical heart resection and left anterior descending artery occlusion myocardial infarct (MI) mouse models [31, 76]. Importantly, there are hints that such an early regenerative window might also exist in newborn humans [2, 32]. This transition from the hypoxic intrauterine environment to the postnatal environment is suggested to cause cardiomyocyte cell cycle arrest. The switch to mitochondrial oxidative metabolism causes a surge in ROS *(reactive oxygen species)* generation [95], which provokes the activation of the DNA damage response, causing cell cycle arrest in postnatal cardiomyocytes, thus inhibiting cardiac regeneration [78]. In support of this, Nakada and colleagues have manipulated the environment of adult mice exposing them to hypoxaemia (manipulated conditions whereby oxygen is gradually decreased by 1% per day until 7% O_2_ is obtained and then maintained for 2 weeks). Strikingly, induction of MI under these conditions resulted in a robust regenerative response with decreased myocardial fibrosis and improved left ventricular systolic functions [68]. In addition to the environmental changes (O_2_ concentration), the development of postnatal endothermy and the associated increase in thyroid hormones have also been proposed as a brake for the cardiac regenerative potential in mammals [36, 70].

Interestingly, the hypothesis that mammalian hearts might have regenerative potential dates back to 1956. In this pioneering study, investigators are able to show how the healing processes in young rats, subjected to a myocardial lesion, proceed rapidly due to high regenerative abilities [83]. However, cardiomyocyte proliferation in the murine heart starts to decrease gradually from embryonic day E8 to E11 as estimated by labeling of DNA synthesis [17]. Since cardiomyocytes cease to proliferate early after birth, the continuous increase in heart mass during postnatal growth occurs through hypertrophy, i.e., the increase in cell size, of pre-existing cardiac myocytes. For instance, in the rat heart, the number of cells increases by 68% in early postnatal days (from day 1 to 3), but remains constant thereafter. Indeed, at 4 days after birth, cardiomyocyte binucleation occurs as a hypertrophic adaptive response to sustain the mechanical load of postnatal development [54]. Of note, several studies observed an increase in cardiomyocyte proliferation after cardiac injury in adult hearts [37, 47, 68]. However, this occurs at very low levels and is insufficient to elicit significant regeneration of the heart [37].

Taken together, these observations confirm that cardiac regeneration in mammals per se is possible. However, it is extremely limited and blunted immediately after birth, but the intriguing possibility to extend regenerative responses to adulthood holds potential for the identification of regenerative treatments for heart failure. In this context, the establishment of in vivo models of complete cardiac regeneration are instrumental for dissecting molecular networks and providing a platform to test novel targeted therapies for the stimulation of cardiomyocyte proliferation and cardiac regeneration in mammals. In the following pages, we provide an overview of the currently available animal models employed to study cardiac regeneration and furthermore summarize their contributions in the identification of molecular and therapeutic approaches for cardiac repair through re-activation of regenerative processes.

## Animal models of cardiac regeneration after injury

In the last decade, scientists focused on determining a highly valid and clinically relevant animal model to study heart regeneration. One major challenge is that the heart is a complex organ that requires the concerted action and interaction of different cell types (predominantly fibroblasts, endothelial cells, and cardiomyocytes among others) to mediate the response to distinct types of myocardial damage [3]. The study of hemodynamic changes, energy metabolism, morphological and functional parameters of the heart in the course of cardiac regeneration and remodelling requires in vivo animal models to recapitulate the pathophysiological features of the heart in the context of the whole organism [20]. Since zebrafish have a remarkable and life-long capacity for complete heart regeneration after severe damage, cryoinjury and ventricular apical resection models in this species have allowed researchers to gain significant insights into cardiac regenerative processes. which can be applied to mammalian models [42]. However, as mentioned above, there are some fundamental differences between fish and mammalian hearts that has therefore led to an increasingly stronger focus on more translatable mammalian models when studying cardiac regeneration.

**Fig. 1:**
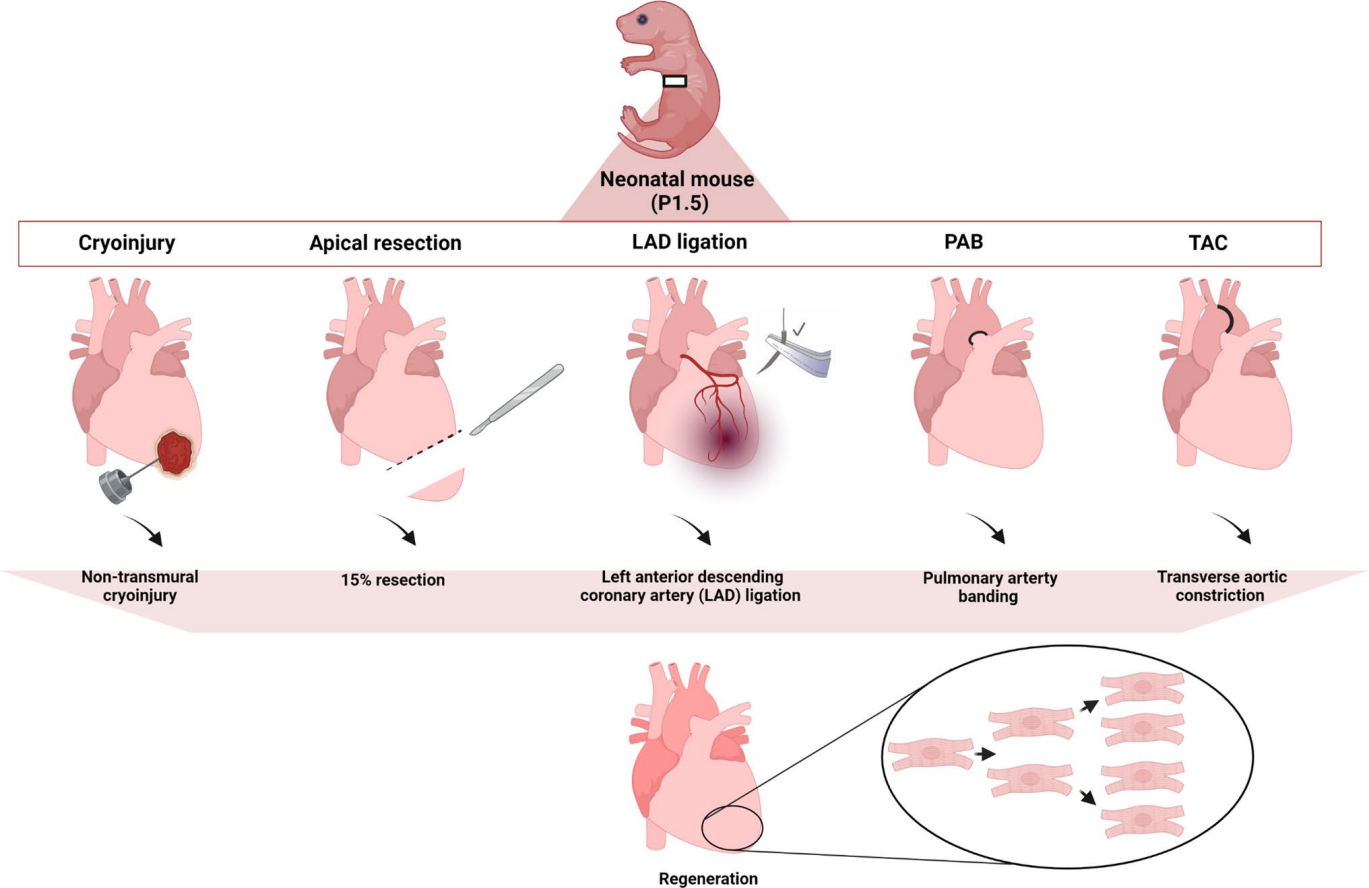
Neonatal cardiac injury models. Cryoinjury is performed through a cryoprobe, resulting in severe and immediate damage to the heart. A part of the left ventricle apex is removed for the apical resection procedure. MI is induced by surgical ligation of the LAD. Non-transmural cryoinjury, apical resection (in the amount 15%) and LAD ligation can trigger full heart regeneration in neonatal mice. Pulmonary artery banding (PAB) surgery allows for cardiac remodeling in a pressure overload system in the right ventricle and proliferation in neonates. TAC is performed by placing a suture under the transverse aorta causing constriction and hypertrophy and left ventricle pressure overload. Common to all models is that the regeneration observed is mainly attributed to the proliferation of pre-existing cardiomyocytes rather than to the differentiation of progenitor cells or transdifferentiation of non-cardiomyocytes. “Created with BioRender.com”

The knowledge of endogenous mechanisms that might regulate the repair and healing of an injured heart in neonatal mice represents a fundamental perspective to stimulate heart regeneration in adult mammals using innate mechanisms. However, a standardized model and a consensus on a method to investigate cardiac regeneration is still lacking. Indeed, three different, challenging, neonatal cardiac injury models are commonly used to investigate cardiac regeneration: the cryoinjury model, the apical resection model and the left anterior descending artery (LAD) ligation model (Fig. 1).

All current experimental injury models are performed under hypothermic anesthesia, which is induced by incubating the neonates in ice water for 2–3 min. Generally, to avoid direct contact with ice, a small barrier is created using a sterile gauze [64]. Successful anesthesia is confirmed by the loss of external reflexes (through the light pinching of the toes and tail). The hypothermia condition induces cardiac arrest, asystole and apnea. While hypothermic anesthesia has no harmful consequences on animal development or cognitive functions in the short and long term [41], it can also be considered a limiting factor for these models, since prolonged time on ice (> 15 min) can drastically increase perioperative mortality. Generally, cardiac injury models require extensive training and excellent surgical skills. which are a prerequisite for the minimization of experimental variability as well as of maternal cannibalism [41, 74].

## The cardiac cryoinjury model

A series of studies on cardiac regeneration have been conducted using a neonatal mouse model of cardiac cryoinjury. First, a transverse skin incision is carried out across the chest, followed by a thoracotomy procedure. Once the heart is exposed, a liquid nitrogen cooled cryoprobe (usually a metal bar with a defined diameter) is applied to the left ventricular surface for a defined period of time. Next, the chest wall is carefully closed by surgical sutures between the ribs of the opened intercostal space and then the cut pectoral muscles are sutured. Lastly, the skin incision is closed with two sutures [33]. Of note, the use of tissue adhesives for skin closure is not recommended as this may strongly increase maternal cannibalism (own observation).

Importantly, Darehzereshki and colleagues have shown that the type and severity of the cryoinjury strongly affect the regenerative responses in neonatal mice. They have developed different cardiac cryoinjury models to induce different degrees of damage, either inducing severe damage by affecting the full wall diameter (transmural), or inflicting mild damage, by not penetrating the entire ventricle wall (non-transmural). A non-transmural cryoinjury in P1 mice can undergo healing without scarring, while hearts with a transmural cryoinjury fail to regenerate. In contrast to other studies employing this model, no statistically significant increase in cardiomyocyte proliferation in either the border or remote areas after transmural or non-transmural cryoinjuries was observed [18]. It is conceivable that the size of cryoablation in neonatal hearts may dictate the degree or lack of complete regeneration, thus limiting the reproducibility of this approach. It should also be taken into consideration when opting for this model that cryoinjury causes a severe and immediate necrotic injury with pathological side effects on the myocardium, which do not recapitulate the ischemic features of human heart disease [33, 73].

## The heart apical resection model

In 2014, Mahmoud and colleagues published a Nature Protocol outlining the apical resection method, in which they described a 10–15 min surgical procedure step by step in neonatal mice at P1 or P7. Following hypothermic anesthesia, a thoracotomy was performed across the lower half of the chest. Once the left ventricular chamber (LV) is exposed, the smallest amount of tissue possible should be removed from the apex of the heart [64]. This represents the most critical step of this technique. Only around 15% should be resected, especially in P7 mice in which the size of the ventricular apical resection strongly influences mortality [99]. The chest closure can then be performed by first suturing the ribs and pectoral muscles together. Skin glue may then be used following internal sutures to reconnect the two ends of the skin incision. However, our own experience indicated that the use of glue significantly increases the rate of maternal cannibalism, which is barely observed when the skin is closed by two surgical knots [33].

In 2011, the pioneers of this method, Porrello and colleagues, have revealed that the surgical resection of 15% of the left ventricular apex in P1 mice is fully restored within 21 days. Immediately after the resection at P1, a robust inflammatory response occurs and the resection site is sealed by a large blood clot. Gradually, the apical clot is resorbed and replaced by normal myocardial tissue. Within 21 days, proliferating cardiomyocytes have substituted the resected tissue and normal cardiac function is restored [76]. The regenerative response is predominantly based on the proliferation of pre-existing cardiomyocytes with only minimal hypertrophy and fibrosis within the regenerated tissue. In 7- and 14-day-old mice subjected to apical resection, heart regenerative processes fail to restore cardiac functions and cannot replace necrotic cardiac tissue [76]. P7 mice are less effective at forming a blood clot to seal the resected tissue.

By contrast, results from Andersen and colleagues are contradictory to those reported by Porrello. In fact, they have claimed that apical resection in P1 mice results in irreversible fibrosis and dilated cardiomyopathy suggesting that regenerative cardiac abilities in mammals are limited, even in the early life stages [3]. They have extended their study from 21 to 180 days post-resection, providing the first long-term functional follow-up study, showing that the regeneration process is incomplete and scarring and cardiac dysfunctions are still substantial 180 days post-resection. They do not exclude that regenerative processes take place following apical resection, but suggest that this occurs at a low level, inadequate to guarantee full heart recovery [4]. The reason for the discrepancy between the studies of Porrello and Andersen are not fully understood, but it may be due to differences in the injury size or due to the different evaluations of the degree of resection. Additionally, the surgical lethality is strictly related to the age of the mice. Similar to cryoinjury, the apical resection model does not recapitulate typical human cardiac damage following an MI. For example, with every additional day after birth, mice have a decreasing capacity to form a blood clot to seal the resection wound, making it very difficult to compare the ongoing regenerative and reparative processes in the neonatal heart.

## The neonatal myocardial infarction mouse model

Left anterior descending coronary artery (LAD) ligation to induce MI in neonatal mice appears to be the most relevant model since it more closely resembles the clinical situation after MI [13]. The pathophysiological aspects of infarction-related myocardial ischemia in humans is faithfully recapitulated in this model. As already described, anesthesia is conducted by hypothermia. The surgery is performed on pups 10–12 h after birth up to 7 days post-partum. The thorax is opened in the fourth intercostal space and the LAD is ligated with a single stitch above the branching point of the vessel with as minimal damage as possible to the cardiac wall [33]. The chest incision is then closed by suturing the ribs and the muscles together. The closure of the skin is then quickly performed using two sutures. The most critical step of this procedure is the visualization of the LAD, likely due to the deep hypothermic anesthesia and consequential cardiac arrest [13]. This aspect combined with the tiny heart size of newborn mice certainly make this technique the most technically challenging cardiac regeneration model compared to an apical resection or cryoinjury. Additionally, the reproducibility of the surgical procedure results in a fluctuation of the infarct size related to the individual anatomy of the animals [13].

Moreover, several critical points can influence the rate of mortality in neonatal mice throughout the surgery and immediately following the operation. Crucial precautions are already required during the anesthesia stage, including the use of melted ice (ice water) to shorten the hypothermia induction time. Moreover, the overall time that the mice are subjected to hypothermic anesthesia induction should not exceed 2–5 min [64]. An additional imperative parameter to take into account is regarding the age of the mice. This needs to be particularly controlled since the recovery, after both anesthesia and surgery, results in more complications for P7 compared to P1 mice [13]. Similar caution is needed both during the ligation of the LAD (as the LAD is extremely delicate and a severe ligation can induce death during surgery) [64] and during the chest closure (as the ribs are extremely fragile and can be easily broken due to the sutures, compromising the success of the operation) [64]. The swift (and successful) completion of the operation is also equally imperative for ensuring the survival of the mice. The hypothermic anesthesia should not exceed 15 min, since longer times will severely lower the survival rate. After the mice awake post-op and are placed back into the mother´s care, it is fundamental to monitor the behavior of the mother to ensure acceptance of the neonates. In fact, a major risk of death after the surgery is associated with maternal cannibalism, particularly if glue is used instead of suturing the skin together [13].

Nevertheless, from a clinical angle, this procedure remains the most relevant for studying pathobiological and regenerative processes. Several studies using this LAD coronary ligation model confirmed the cardiac regenerative potential after MI at P1 through compensatory cardiomyocyte proliferation [32, 77]. In contrast, Konfino et al. observed only incomplete cardiac regeneration following LAD ligation in mice at P1 [49].

## Novel neonatal models of cardiac stress provoked by large artery constriction

Notably, the pressure overload model by pulmonary artery banding (PAB) surgery is a recent addition for studying heart regeneration in the neonatal rat. Investigators suggest that this model is quite exhaustive to investigate cardiomyocyte proliferation. Differentially expressed genes between cardiomyocytes from PAB and sham-operated rats are mainly implicated in mitosis and cell division. Additionally after PAB, stress may extend the period of cardiomyocyte proliferation beyond 7 days after birth. The rate of cardiomyocyte proliferation is increased in rats after PAB surgery, concluding that the pressure overload can induce the heart to proliferate at the neonatal stages. Thus, this model may be useful to study congenital heart diseases, such as pulmonary stenosis, tetralogy of Fallot and other chronic heart failure diseases [103].

Similarly, Mahammadi et al. have established a transverse aortic constriction protocol in neonatal mice (nTAC) in the regenerative (at P1) and non-regenerative stages (at P7). The TAC model is commonly used in adult mice to investigate cardiac pressure overload-induced left ventricular heart failure. However, Mahammadi and colleagues aimed to compare potential regenerative responses of neonatal mice to nTAC surgery in P1 versus P7. Their results revealed that nTAC at P1 induces an adaptive response, preserving cardiac function and increasing cardiomyocyte proliferation within the regenerative window phase. In contrast, nTAC in P7 mice, in which the cardiomyocyte proliferation capacity is lost, causes cardiomyocyte hypertrophy, fibrosis and cardiac dysfunction. The authors suggest that the adaptive regenerative response toward pathological pressure overload triggers cardiomyocyte proliferation immediately after birth, thus mimicking physiological hypertrophy that occurs during pregnancy or during sport. In contrast to preserved cardiac function, nTAC outside of the regenerative window is accompanied by cardiac dysfunction and fibrosis, which is typical for pathological hypertrophy as observed in the adult heart exposed to chronic pressure overload [65, 67].

Extra care and exceptional technique are required in the performance of both PAB [96] and TAC surgeries to avoid damaging arteries in the extremely fragile neonatal hearts. Additionally, these two surgical techniques in neonates could also result in improved models for myocardium remodeling in addition to studying cardiac regenerative processes.

## Molecular basis of heart regeneration and potential targets for clinical translation

The common concept of the described cardiac regeneration models is to identify molecular switches that promote cell cycle re-entry and mitosis of resident cardiomyocytes, which upon injury can proliferate and replace damaged tissue to maintain cardiac functions. In this section, we highlight recent studies utilizing neonatal cardiac regeneration models to identify factors that could be successfully exploited to induce cardiac regeneration in adult cardiac injury models. Our purpose is to provide a general overview of potential therapeutic targets for enhancing endogenous cardiomyocyte proliferation that may have the potential for translation into larger animal models and potentially humans (see Table 1).

**Table 1:** List of the most promising molecular targets identified in promoting heart repair

Molecular factor	Zebrafish model	Neonatal mouse model	Adult mouse model	Large animal model	References
**Hypoxia**	Yes, ventricular amputation of zebrafish heart Hyperoxia and dnHIF1a overexpression	Yes Neonatal mice are exposed to hyperoxic and hypoxic environments	Yes, MI model Exposure to very low oxygen concentrations	No documented evidence	Jopling et al. (2012) [43] Puente et al. (2015) [78] Nakada et al. (2017) [68]
**Gata4**	Yes, transgenic zebrafish Gata4 overexpression in cardiomyocytes of transgenic zebrafish	Yes, neonatal cryoinjury model Yes, neonatal TAC model Studies on Gata4 knock-out mice after injury	Yes, MI model Assessment of ventricular function and fibrosis formation after administration of Gata4 in rat	Yes Studies in two patients in families with the Gata4 gene mutation	Garg et al. (2003) [26] Singh et al. (2009) [86] Mathison et al. (2017) [66] Karra et al. (2018) [45] Mohammadi et al. (2017; 2019) [65, 67]
**Telomerase**	Yes, cryoinjury model Studies on Tert knock-out zebrafish model	Yes, neonatal cryoinjury model Studies on Tert knock-out mice	Yes, MI model Tert re-activation using AAV gene therapy	Yes, MI model in pigs Quantitative evaluation of telomerase activity	Bednarek et al. (2015) [10] Aix et al. (2016) [1] Bär et al. (2014) [8] Zhu et al. (2018) [105] Chatterjee et al. (2020) [15]
**Meis1**	No specific studies	Yes, MI model Studies on neonatal mice overexpressing Meis1	Yes Studies on tamoxifen-inducible knock-out mice	No documented evidence	Mahmoud et al. (2013) [63]
**NRG1/ERRB2**	Yes Loss- and gain- of-function genetic experiments	Yes, neonatal cryoinjury model Early administration of *NRG1*	Yes, MI model Loss- and gain-of-function genetic experiments	Yes, preclinical studies in canine model of pacing-induced heart failure Recombinant human Nrg1 administration	Liu et al. (2006) [58] Gemberling et al. (2015) [27] Polizzotti et al. (2015) [73] D`Uva et al. (2015) [20] Gao et al. (2010) [24]
**Macrophage**	Yes, cryoinjury model Delayed macrophage recruitment	Yes, MI model Macrophage-depleted mouse model	Yes, MI model Inhibition of monocyte recruitment	No documented evidence	Lai et al. (2017) [51] Aurora et al. (2014) [7] Lavine et al. (2014) [52] Li et al. (2021) [56]
**Tregs**	Yes, cryoinjury model Treg cell depletion in zebrafish	Yes, neonatal apical resection and cryoinjury model Studies on neonatal NOD/SCID mice	Yes, MI model Treg soluble factors overexpression using AAV gene therapy	No documented evidence	Hui et al. (2017) [39] Li et al. (2019) [55] Zacchigna et al. (2018) [104]
**Agrin**	No specific studies	Yes Loss and gain of functions	Yes, MI model Recombinant agrin administration	No documented evidence	Bassat et al. (2017) [9]
**YAP**	Yes, cryoinjury model Yap deletion in adult zebrafish	Yes, MI model Loss of function	Yes, MI model Human Yap overexpression using AAV gene delivery	Yes, MI model in pig AAV-based therapy to knock-down Hippo pathway	Xin et al. (2013) [98] Lin et al. (2014) [57] Flinn et al. (2019) [22] Liu et al. (2021) [61] Liu et al. (2021) [60]
**Thyroid hormones**	T3 (triiodothyronine) treatment in adult zebrafish	Blocking of thyroid hormone synthesis → increasing cardiomyocyte proliferation	Yes, MI model Studies on mutant mice for thyroid hormone receptor	No documented evidence	Hirose et al. ((2019)) [36]

## Molecular targets promoting regenerative processes

Using the different mouse models described above, several molecular targets with the potential to enhance cardiac regeneration beyond the very early postnatal stages were identified to date. One conceivable target is the expression of telomerase based on its intimate association with the maintenance of telomere length, stem cell renewal, and senescence [5, 12]. Regeneration competent fish and neonatal mouse hearts actively express telomerase, whereas the loss of cardiac regeneration in neonatal mice coincides with the silencing of telomerase expression. Indeed, Bednarek et al. showed that the ability to regenerate after cardiac cryoinjury is lost in telomerase deficient zebrafish [10]. In contrast, therapeutic activation of telomerase in adult mice by means of gene therapy utilizing adeno-associated viruses (AAV) was protective after induction of MI by LAD ligation [8, 15]. Improved cardiac function, reduced infarct scar size and increased survival could at least partially be explained by increased numbers of proliferating cardiomyocytes in the infarct border zone [8].

Using the cardiac cryoinjury model in newborn mice, Mohammadi and colleagues uncovered that the overexpression of the transcription factor *Gata4* enhances cardiac regeneration as evident by reduced scar formation concomitant with increased cardiomyocyte mitosis, cell division, and angiogenesis [65].

Another cryoinjury study in newborn mice has been conducted to assess the role of Neuregulin-1 (NRG1) in cardiac regeneration. Early administration of recombinant NRG1 prevents transmural scar formation leading to a better outcome of cardiac functions and stimulates cardiomyocytes cell cycle re-entry and proliferation in the first 10 days of life [73]. To determine whether such a stimulatory function is present also in humans, the authors have isolated cardiomyocytes from human pediatric patients with cardiac disease that underwent heart surgery. It was seen that NRG1 was involved in promoting cardiomyocyte cycling in hearts from infants who are less than 6 months of age [73]. In another report, using loss- and gain-of-function genetic experiments in mice, others have revealed that NRG1/ERRB2 signaling takes part in the control of cardiomyocyte proliferation. Specifically, they have demonstrated that transient expression of ERRB2 after LAD ligation either in juvenile or in adult hearts triggers cardiomyocyte de-differentiation and proliferation leading to regeneration [20].

Another study has demonstrated the critical role of Meis1 as a master transcriptional regulator of the cardiomyocyte cell cycle. Cardiac-specific Meis1 overexpression inhibits neonatal heart regeneration following MI at P1 via the induction of the cyclin-dependent kinase (CDK) inhibitors p15, p16, and p21. In contrast, cardiomyocyte-specific inactivation of Meis1 in adult mice by using tamoxifen-inducible knock-out mice (αMHC-MerCreMer mice) stimulates cardiomyocyte cell cycle re-entry and re-activation of mitosis [63].

Inflammatory responses and the involvement of bone marrow-derived and cardiac resident immune cells have emerged as crucial factors in the healing of the heart following MI [19, 30, 84]. Acute MI leads to cellular, molecular and functional alterations that trigger immune responses. Aurora and colleagues hypothesized that macrophages, which are recruited abundantly to the infarction zone, may not only have a role in infarct healing but also in regeneration [7, 72, 84]. Using a macrophage-depleted mouse model, they demonstrated that P1-depleted mice following MI are unable to have heart tissue regeneration, showing instead a reduced cardiac function and fibrotic scar formation [7, 56]. Thus, modulation of inflammatory responses may provide a key therapeutic strategy to support heart regeneration [30]. Nevertheless, there seems to be a fine balance: while inflammation is needed early after MI, it must also be contained to avoid exaggerated remodeling of the damaged heart [44]. Regulatory T cells (Tregs) are involved in the suppression of inflammatory responses and in the resolution of inflammation after tissue injury. Indeed, Tregs were recently discovered to participate in the regulation of heart regeneration. For example, Li et al. demonstrated that NOD/SCID mice that harbor innate immune cells such as macrophages, but lack functional adaptive immune cells such as T cells, fail to have regeneration of their heart after apical resection or cryoinjury in P3 mice. In contrast, adoptive transfer of Tregs results in mitigated fibrosis and enhanced proliferation and function of the injured myocardium [55]. In addition, Zacchigna et al. have described the beneficial effects of Treg soluble factors in improving the outcome of ischemic heart disease. Injection of Tregs in the mouse heart reduces infarct size, preserves contractility, and increases the number of proliferating cardiomyocytes. Importantly, cardiomyocyte-specific therapeutic overexpression of six factors secreted by Tregs (Cst7, Tnfsf11, Il33, Fgl2, Matn2, and Igf2) phenocopied the beneficial effects after MI [104].

Importantly, Bassat and colleagues highlighted a critical role of the extracellular matrix for cardiac regeneration. Agrin, an endothelial-derived extracellular matrix component highly expressed at birth, is required for regeneration in neonatal hearts. Treatment of adult mice with recombinant agrin promotes cardiac regeneration after an MI. Mechanistically, the binding of agrin to its receptor, Dag1, induces myofibril disassembly and activation of Yes-associated protein (YAP), a well-described inducer of cardiomyocyte proliferation [9] (Table 1). In line with this, in vitro treatment of human-induced pluripotent stem-derived cardiomyocytes (hiPSC-CMs) with agrin prevents their maturation and promotes proliferation [9].

Further studies have underlined a prominent role of YAP as a regulator of cardiomyocyte proliferation. YAP is a terminal effector of the Hippo pathway. Its activation inhibits the Hippo pathway and results in promoting cardiomyocyte proliferation and heart functional recovery both in murine and porcine models of MI [57, 60]. The critical role of YAP in cardiac regeneration can also be exerted by promoting the WNT pathway activation [61, 98].

While these are some of the most promising strategies, for which the neonatal cardiac injury models have been instrumental, the number of protein-coding genes that proved effective after manipulated for cardiac regeneration in the adult heart remains relatively small. In recent years, a new molecular class, namely non-coding RNAs, particularly microRNAs (also known as miRs or miRNAs), have emerged as master regulators of many biological processes and have already been explored in the field of regenerative medicine.

To validate their role, several of these targets have been tested in vitro and in neonatal and adult mouse models in vivo. A few of these targets were also evaluated in large animal models. Mostly, these molecular factors act by stimulating regenerative processes and cardiomyocyte proliferation, but others can promote heart healing by exercising their role on other processes (e.g., fibrosis; congenital heart defects).

## MicroRNAs, regulators of cardiac gene expression and fine-tuners for regenerative processes

**Fig. 2:**
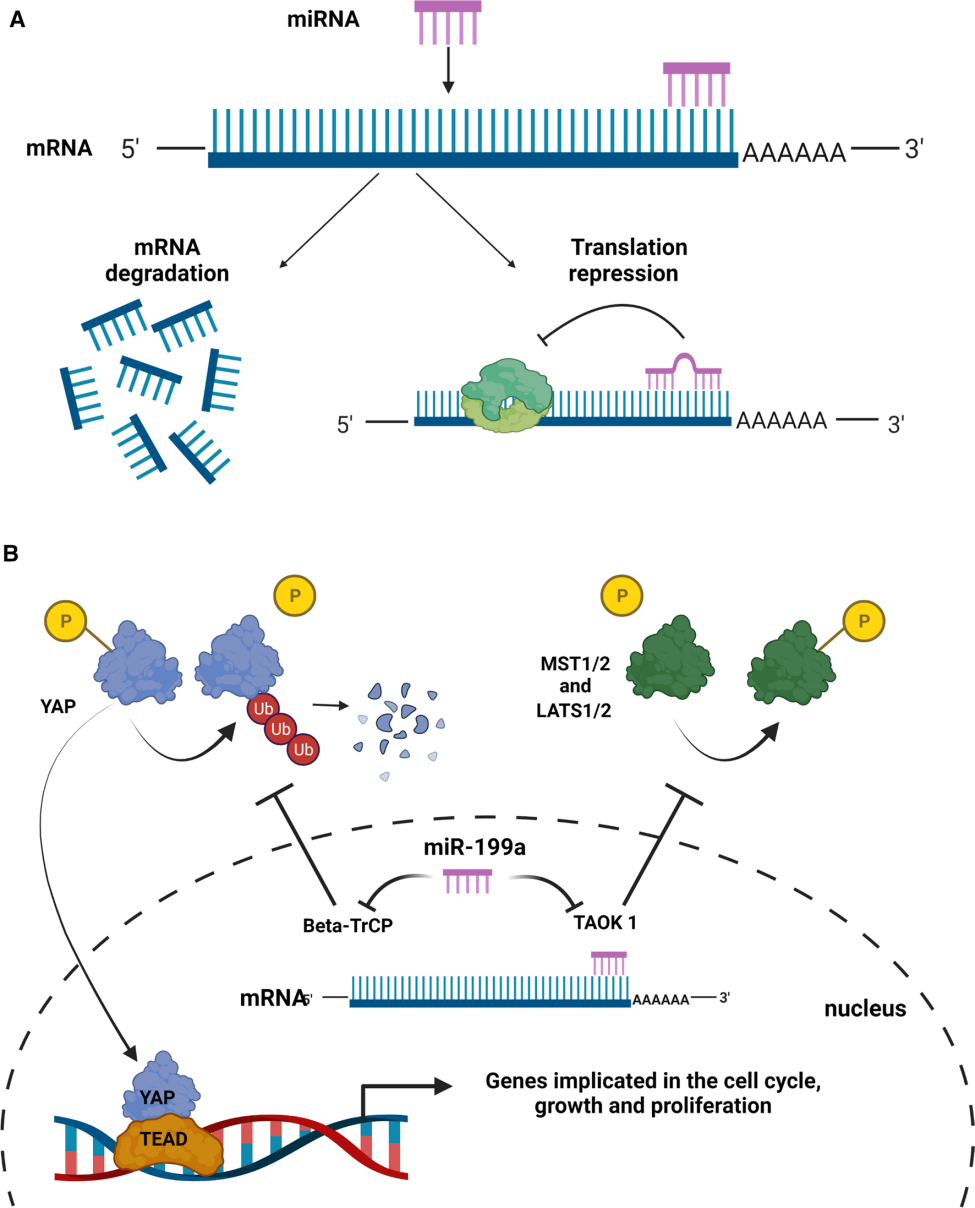
MicroRNA modes of actions. **A** MiRNAs bind mRNA targets at the 3` UTR by base pairing. Targets are inhibited through translational repression or by mediating degradation, leading to specific regulation of gene expression levels. **B** miR-199a as an example of a possible mode of action for a miRNA. miR-199a downregulates the mRNA levels of TAOK1 (TAO kinase 1) and beta-TrCP (beta-transducing repeat containing protein). TAOK1 targets MST1 and LATS1/2, while beta-TrCP promotes the dephosphorylation of YAP and the subsequential degradation. By inhibiting TAOK1 and beta. TrCP, miR-199a promotes the nuclear translocation of YAP, its binding with TEAD (transcriptional enhanced associated domain) and the regulation of target genes involved in the cell cycle, growth and proliferation. “Created with BioRender.com”

MicroRNAs are a class of small regulatory molecules with a short size of 20–23 nucleotides. MicroRNAs mainly interact with the 3´ untranslated region (3´ UTR) of target messenger RNAs (mRNAs) through complementary base pairing; and very seldom are also interacting with the 5` UTR or coding gene sequences [69]. Their binding regulates gene expression at the post-transcriptional level either by promoting degradation or by inhibiting translation of the target mRNA [91] (Fig. 2).

Here, we focused on microRNA-based therapies, which stimulate cardiomyocyte proliferation after cardiac injury in mice and result in promising preclinical studies. The first evidence for miRNAs playing a role in cardiomyocyte proliferation was provided by Eulalio et al. Their functional high-throughput screening for 875 human microRNAs in neonatal rat cardiomyocytes identified 204 microRNAs that significantly increased cardiomyocyte proliferation. Two of the most potent miRNAs regarding the induction of cardiomyocyte proliferation in vitro (miR-590 and miR-199a) were selected and further tested in vivo in mice. Neonatal mice are injected with AAV9 virus particles, overexpressing miR-590 or miR-199a, both resulting in an increased number of dividing cardiomyocytes. Infarcted hearts of adult mice (8–12 weeks), injected at the time of LAD ligation with AAV9 vectors expressing the two miRNAs, showed noticeable cardiac regeneration and almost complete recovery of cardiac functional parameters. A search for the relevant targets of these two miRNAs, revealed genes primarily belonging to the cell cycle, cell growth, and proliferation pathways (e.g., Homer1, Hopx, Clic5, etc.) [21]. Furthermore, they have confirmed the potential of these two miRNAs to stimulate cardiac repair after MI in adult mice after intra-cardiac delivery by means of lipid particles as carriers for synthetic miRNAs [53].

Likewise, a group of miRNAs, miR-17-92, have been investigated for their critical role in postnatal cardiomyocyte mitotic arrest. Deletion of the miR-17-92 cluster in neonatal mice caused a decrease in cardiomyocyte proliferation, whereas overexpression in cardiac-specific transgenic mice (3-week-old) induced cardiomyocytes proliferation. In a disease setting, the effect of miR-17-92 overexpression in tamoxifen-inducible adult mice (4-month-old) subjected to MI by LAD ligation resulted in preserved cardiac functions accompanying an induction of cardiomyocyte proliferation. Mechanistically, miR-17-92 targets PTEN which in turn mediates the regulation of cardiomyocyte proliferation [16]. Later, similar results pinpointed to miR-19a/19b, a member of the miR-17-92 cluster, which is sufficient to enhance cardiomyocyte proliferation and stimulate cardiac regeneration following MI [25].

Porrello and colleagues have elucidated the role of the miR-15 family in repressing cardiac regenerative capacity in mammals after birth. Multiple members of the miR-15 family are up-regulated in mice after birth. MiR-195 (a member of the miR-15 family) transgenic mice subjected to LAD ligation at P1 showed extensive myocardial necrosis and inhibition of cardiomyocyte proliferation. Administration of LNA (locked nucleic acid) modified miR-15 anti-miRs to P1, 7 and 14 mice, followed by LAD ligation at P21 indeed stimulated cardiomyocyte proliferation and promoted myocardial regeneration in adulthood [75, 77]. Yang et al. have investigated the function of miR-34a in promoting cardiac regeneration in mice after MI and have shown that miR-34a overexpression in the neonatal heart inhibits cardiac regeneration, whereas its suppression in the adult heart increases cardiomyocyte proliferation and improves cardiac functions post-MI. MiR-34a modulates cardiac regeneration by targeting Sirt1, Bcl2 and cyclin D1, which are involved in pro-proliferation and pro-survival events [101]. Similarly, miR-128 has been identified as a critical regulator in controlling endogenous cardiomyocyte proliferation. Transgenic miR-128 expression in neonatal mice (P1) presents with left ventricular dilatation and defective regeneration 21 days after apical resection. Conversely, subjecting adult tamoxifen-induced miR-128 knock-out mice to MI leads to an increase of Aurora-B-positive cardiomyocytes, reduced cardiac fibrosis, and robust cardiac regeneration [38].

Several miRNAs are involved in the withdrawal from the cardiomyocyte cell cycle and in the regulation of cardiomyocyte proliferation. Here, we have summarized the most common miRNAs studied for cardiac regeneration (see Table 2). Among these, there are miR-133a, miR-29a, and miR-302–367 clusters and several others which play critical roles in modulating proliferative and apoptotic processes of cardiomyocytes [14, 59, 92, 100]. In summary, translation of such miRNA-based therapies might represent a powerful therapeutic approach to promote cardiac repair and boost regenerative medicine. Indeed, the first worldwide miRNA-based therapy has revealed that an antisense oligonucleotide therapy can attenuate and even reverse heart failure in human preclinical models [90].

**Table 2:** List of the most promising miRNAs, which play a crucial role in cardiac regeneration

miRNA	Neonatal mouse model	Adult mouse model	Gene targets	Effect on cardiomyocyte proliferation	References
**miR-199a** **miR-590a**	No cardiac injury models AAV injection in neonatal mice	Yes, MI model Intra-cardiac delivery of cationic lipid particles and AAV injection in the heart at the time of the injury AAV gene therapy in infarcted pig	Genes implicated in cell cycle, growth and proliferation (Homer1, Hopx, Clic5)	Overexpression → induction of cardiomyocyte proliferation	Eulalio et al. (2012) [21] Lesizza et al. (2017) [53] Gabisonia et al. (2019) [23]
**miR-17-92 cluster** **miR-19a/19b**	No cardiac injury models Studies on transgenic and knock-out mice	Yes, MI model Overexpression in tamoxifen-inducible adult mice subjected MI and using AAV gene therapy	PTEN	Overexpression → induction of cardiomyocyte proliferation	Chen et al. (2013) [16] Gao et al. (2019) [25]
**miR-15** **miR-195**	Yes, MI model Studies on transgenic mice overexpressing mi-195 and LNA anti-miR administration	Yes, MI model LNA anti-mir administration in P 1–7 and 14-day-old mice	Genes implicated in cell cycle regulation Chek1	Inhibition → induction of cardiomyocyte proliferation	Porrello et al. (2011, 2013) [75, 77]
**miR-34a**	Yes, MI model miR-34a inhibition	Yes, MI model miR-34a overexpression	Sirt1, Bcl2 and CyclinD1	Inhibition → induction of cardiomyocyte proliferation	Yang et al. (2015) [101]
**miR-128**	Yes, apical resection model Studies on conditional transgenic neonatal mice overexpressing miR-128	Yes, MI model Studies on tamoxifen-inducible miR-128 knock-out mice	Suz12	Inhibition → induction of cardiomyocyte proliferation	Huang et al. (2018) [38]
**miR-133**	No cardiac injury models Studies on knock-out or transgenic embryonal and neonatal mice	No cardiac injury models Studies on knock-out and transgenic mice overexpressing miR-133 mice	Regulation of the appropriate levels of SRF and CyclinD2	Loss → causes cardiac dysfunction and aberrant excessive cardiomyocyte proliferation and apoptosis Overexpression → diminishing of cardiomyocyte proliferation	Liu et al. (2008) [59]
**miR-29a**	Primary cell studies	Primary cell studies	CyclinD2	Inhibition → induction of cardiomyocyte proliferation	Cao et. al. (2013) [14]
**Cluster** **miR-302–367**	Yes Gain-of- function experiments	Yes, MI model Conditional mice overexpressing miR-302–367 cluster	Inhibition of the Hippo pathway activity through repression of the kinases Mst1, Lats2, and Mob1b	Overexpression → induction of cardiomyocyte proliferation	Tian et al. (2015) [92] Xu et al. (2019) [100]

To validate their role, several of them have been investigated in vivo by using genetic-modified mice models.

## Translational perspectives

**Fig. 3:**
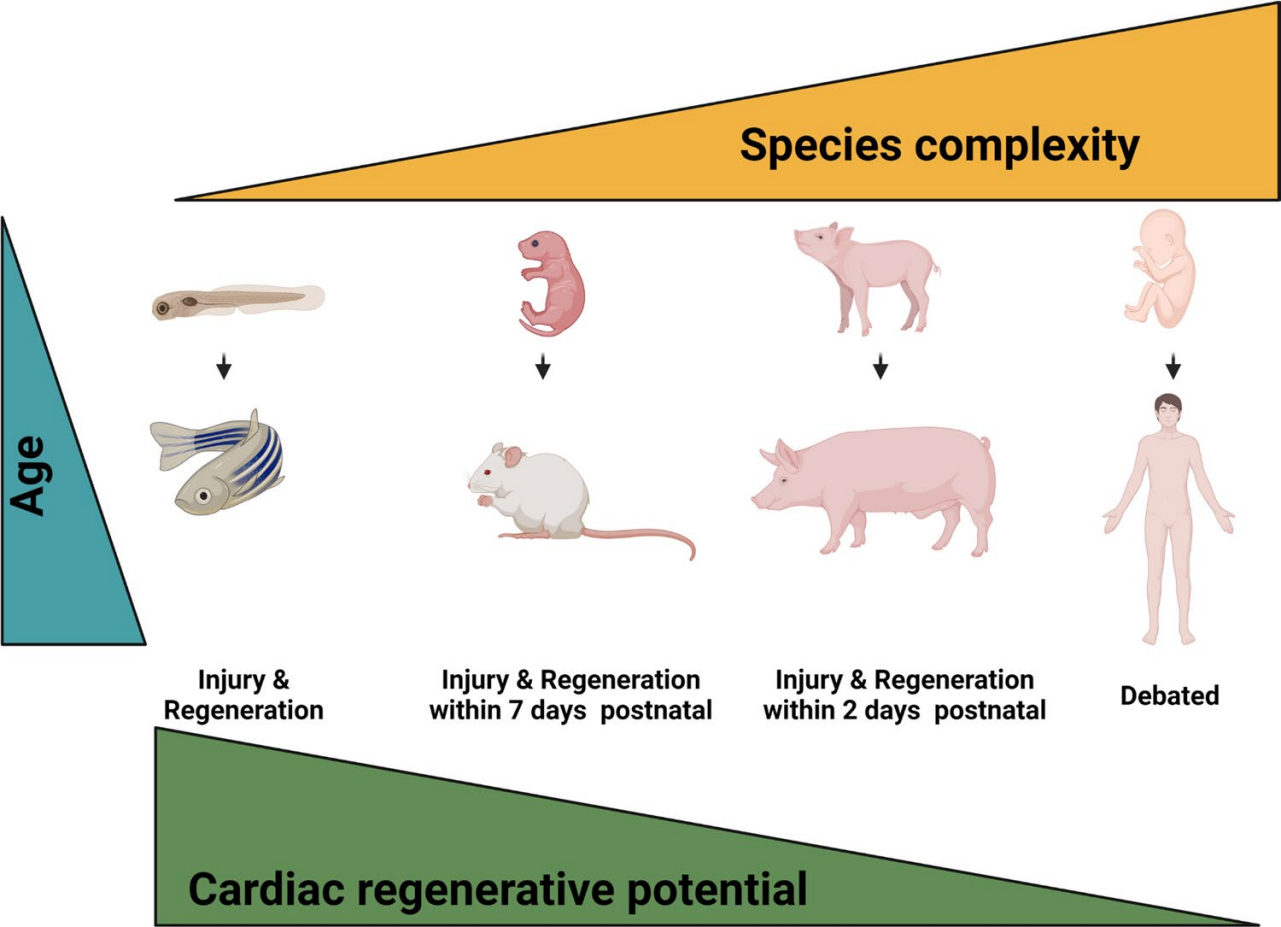
Cardiac regenerative potential in animal models. In response to injury, zebrafish can regenerate the heart without scarring within 2 months as well as in adulthood. Embryonic and neonatal mice retain the capacity to regenerate the heart after injury, but this ability is lost at 7 days postnatal, and in adult mice, thereafter the heart undergoes adverse and pathological remodeling after injury with fibrotic scar formation. Recently, important insights are gained in large mammalian animals. The hearts of neonatal pigs are capable of regeneration during the first 2 days of life. The human heart may have a similar capacity to other mammalians to regenerate, but the heart regenerative potential in humans needs still to be explored. “Created with BioRender.com”

There is still much to learn about cardiac regeneration, particularly, how the insights gained in neonatal studies can help uncover the regenerative potential of the adult heart and in the future, to translate this knowledge to humans (Fig. 3).

Significant efforts are being made into the investigation of cardiac regeneration to both accurately and precisely establish a faithful system to determine cardiomyocyte turnover. To assess cardiomyocyte renewal, investigators performed colorimetric assays and immunostaining for proliferation markers on heart tissue sections. One major challenge is that cardiomyocytes that undergo bi-/polynucleation or endoreduplication stain positive for commonly used proliferation markers such as Ki67 or phospho-Histon3 in the absence of true proliferation [94]. This limitation can be overcome by utilizing elegant transgenic mouse models, which can unequivocally identify cardiomyocytes that underwent cell division [81, 85].

The “era” of hiPSC-CMs has additionally allowed for the development of an innovative tool to track and monitor the fate of proliferating cardiomyocytes, resulting in a directly translational platform for human studies. However, due to the lack of a perfect model, several unresolved issues have currently been identified in vitro (for instance, limitations due to the immature phenotype of hiPSC-CMs). Therefore, major investigations in vivo have been conducted in rodents, which more closely resemble a complete disease model than an individual cell type, but can still maintain a variable comparability to the exact manifestations of complex human diseases. To face these challenges, the findings from studies in murine models need to be projected to large mammal models which are anatomically and physiologically more similar to humans.

In their study, Kim and colleagues have shown that the timing of regenerative arrest is linked to the cell cycle exit and polynucleation of cardiomyocytes. Their studies in preterm (~ 3 weeks before birth) piglets underline that ~ 10% of cardiomyocytes are binucleated, smaller, proliferative with only a few undergoing apoptosis, while on the contrary, in term (2 day before birth) piglets, the rate of binucleated cardiomyocytes increases to ~ 30% [46]. More recently, a neonatal porcine heart model of MI was used for cardiac regeneration studies. Ye and colleagues have provided an important report on regenerative capacity of injured porcine heart at the ages of 2, 3 or 14 days postnatally by using a permanent coronary artery ligation (LAD) approach similar to the methods described in neonatal mice. The cardiac muscle can be regenerated in 2-day-old pigs following MI resulting in the absence of scar fibrotic tissue and with a spectacular improvement in cardiac function [102, 105]. This scenario can be attributed to cardiomyocyte cell cycle re-entry, proliferation and cytokinesis. In contrast, cardiac injury after day 2 cannot be regenerated, concluding that the heart of large mammals has indeed capacity for regeneration, but this potential is lost very rapidly after birth within 2 days [102]. Further evidences has shown that the rapid decline of cardiomyocyte cell cycle activity is accompanied by a decline in telomerase activity, meaning that cardiomyocytes that have exited the cell cycle have shorter telomeres. This finding paves the way to speculate that telomerase may play a crucial role in the stimulation of cardiomyocyte proliferation, consistent with the evidences acquired in neonatal and adult mice, already described above. Other important results are been achieved in 3-month-old pigs in which the contribution of the human miR-199a in cardiac repair was investigated. AAV9-miRNA-199a injection in infarcted pig hearts contributed to the endogenous stimulation of cardiomyocyte proliferation resulting in a functional recovery after injury. Nevertheless, this treatment needs careful controlling of the therapeutic dosing to avoid persistent overexpression, which can lead to uncontrolled proliferation and formation of heart tumors [23]. Today, the challenge is to develop a safe and efficient gene delivery system that specifically targets cardiomyocytes. Recently synthetic modified mRNAs (modRNAs) have been developed as an innovative gene delivery method that is largely used in in vivo studies after MI [29]. In contrast to AAV vectors, a cardiomyocyte-specific modRNA delivery enables the transient induction of cardiac regenerative processes in the injured myocardium and stimulates endogenous cardiomyocyte proliferation and reintroduction into the cell cycle in a rather controlled fashion based on the short half-life of therapeutic RNA molecule [62].

Whether the human heart possesses a similar regenerative capacity as mice or pigs remains unknown. A recent review highlighted a very early study of Wesselhoeft et al. with congenital pediatric heart disease that causes myocardial ischemia, named *ALCAPA* syndrome (anomalous left coronary artery from the pulmonary artery) [97]. They have demonstrated how a cardiac surgery approach within the first year of age leads to complete recovery of the heart, whereas delayed diagnosis most often ends in damage and ultimately ischemic cardiomyopathy [34]. Furthermore, Haubner and colleagues have investigated the mechanisms of cardiac regeneration in humans in an interesting case study on a newborn child with a severe perinatal infarct due to coronary artery occlusion. The patient´s heart recovers completely one-and-a-half months after birth showing apparently normal cardiac functional and structural parameters despite delayed thrombolysis reperfusion therapy [32]. These results suggest that similar to neonatal animal models (e.g., murine and porcine), newborn humans might retain the capacity to repair cardiac damage by endogenous proliferative processes resulting in the replacement of necrotic tissue with new cardiomyocytes. Another interesting and surprising case in humans was performed on an 11-month-old girl with severe heart failure. She was subjected to a heterotopic cardiac transplantation (using a donor heart from a patient of 5 months placed in an ectopic position without removing the native heart). Unexpectedly, 10.5 years after the original operation, the native heart has shown full recovery and normal cardiac function allowing the removal of the donor heart. These examples further highlight the capacity of young human hearts to be repaired [93]. Furthermore, in a recent study, a rare case of a female child born by a diabetic mother suffered a myocardial infarction confirmed by echocardiography and serum diagnostic parameters. The newborn patient was treated with inotropic support, diuretics and afterload-reducing agents, such as beta-blockade, with the necessary caution for the application in a newborn. Strikingly, the child recovered complete myocardial functions with normalization of ventricular systolic and diastolic function and was asymptomatic at the age of 3 years [2]. These studies support the hypothesis for cardiomyocyte proliferative activity as a driver for regenerative repair of the heart in the early postnatal time. However, crucial issues remain to be clarified: first, can the mechanisms of cardiac regeneration in neonates be transferred to the adult heart, and second, how can cardiomyocyte proliferation in adult human hearts be induced in a controlled and safe manner.

## Conclusions

Presently, several strategies for regenerating the injured and diseased heart are pursued. In addition to stimulating endogenous cardiomyocyte proliferation, transdifferentiation of non-cardiomyocytes, e.g., cardiac fibroblasts that constitute the fibrotic infarct scar, into cardiomyocytes is a possibility that is currently being explored [87]. However, despite regenerative responses induced in the injured adult heart by applying factors identified in neonates, these strategies are still exclusively limited to the preclinical research state. This is primarily based on the complex differences between rodent and human hearts, the (non)-transferability of findings from the rather plastic to the terminally differentiated adult heart, as well as several hurdles concerning delivery and safety of factors that induce proliferation in the human body. Nevertheless, we are convinced that continuous research will lead to the development of endogenous, regenerative strategies in the clinics, although there is still a long way to go and progress will probably only be made in small increments. Lessons can certainly also be learned from (stem) cell therapy approaches to regenerate the heart, which currently represents the predominant strategy that is already investigated in a number of clinical trials [80, 82]. However, several concerns such as stem cell fate and engraftment, dosage, and mode of administration exist and remain open and unresolved [82]. Although some encouraging results show cardiac functional improvement, even after decades of intensive research, cell-based therapies are also far from becoming clinically routine [88].

To conclude, despite the numerous insights into cardiac regeneration using fascinating animal models, the regenerative potential of the heart needs further elucidation and tremendous efforts are still required to develop innovative and safe therapies to be translated from experiments to treatments for patients with cardiac diseases.
